# Synthesis of LiNbO_3_ nanoparticles in a mesoporous matrix

**DOI:** 10.3762/bjnano.2.3

**Published:** 2011-01-10

**Authors:** Anett Grigas, Stefan Kaskel

**Affiliations:** 1 Department of Inorganic Chemistry, Dresden University of Technology, Mommsenstraße 6, D-01062 Dresden, Germany

**Keywords:** LiNbO_3_, ordered mesoporous material, SBA-15, template synthesis

## Abstract

The synthesis of LiNbO_3_ nanoparticles in SBA-15 is reported for the first time. The preparation of nanoparticles was carried out by impregnation from the metal precursor and mesoporous silica SBA-15 as the template. A rapid one-step treatment in an IR furnace reduces the preparation time to only 10 min. In comparison, a conventional furnace requires 5 h reaction time to produce nanoparticles with similar textural properties. Another advantage of applying an IR furnace compared to conventional heating is the extremely high heating rate (up to 10 °C/s) and corresponding time saving. The resulting samples were investigated by powder X-ray diffraction, nitrogen physisorption, and high resolution transmission electron microscopy (HRTEM). The obtained nanoparticles are spherical with a diameter of approximately 10 nm.

## Introduction

Lithium niobate is one of the most important ferroelectric materials. At room temperature it has a rhombohedral symmetry and space group *R*3*c* [1]. It is well known that LiNbO_3_ has excellent piezoelectrical, pyroelectrical, electro-optical and nonlinear optical properties [2–3]. As a result, there are many valuable applications, for example, in integrated optics or surface acoustic wave devices [4–5].

In the past decade, several methods for the synthesis of LiNbO_3_ have been investigated such as the Czochralski method [6–7], the sol–gel route [8–11], and hydrothermal treatment [12–14]. Of all these synthetic approaches, only the hydrothermal treatment is suited for the preparation of nanosized particles due to the mild reaction conditions involved [15]. Another ingenious route to generate nanoparticles is the use of porous template materials via impregnation techniques, where the oxide is obtained by calcination of suitable precursor solutions with simultaneously removal of the carbon matrix [16–18].

In the present work, we investigated a rapid and efficient one-step route for the direct IR-accelerated synthesis of LiNbO_3_ nanopowders using an ordered mesoporous silica SBA-15 as the template, in combination with an IR furnace for the crystallization. The matrix stabilizes the nanoparticles against sintering while the heat treatment promotes crystallization. For material tailoring purposes the factors influencing the particle size under the reaction conditions on the size of the nanoparticles were considered. The IR furnace uses a lamp for indirect heat treatment and the wavelength can be tuned with the choice of the lamp. In the focal line of a gold plated cylinder with an elliptic profile, resides an IR radiator focusing the radiation in the second focal line where the sample is located inside a quartz tube flow reactor. Temperature measurement and control is carried out via a NiCr-Ni thermocouple located close to the sample.

## Results and Discussion

In general for the preparation of LiNbO_3_, SBA-15 was impregnated with a solution containing LiNO_3_ and NH_4_NbO(C_2_O_4_)_2_·*x*H_2_O. Crystallization was carried out in air using an IR furnace IRF 10 (Behr) in the temperature range 750–1000 °C (see Experimental section). LiOH solution was used to remove the SBA-15 matrix and the isolated product was air-dried.

**Figure 1:**
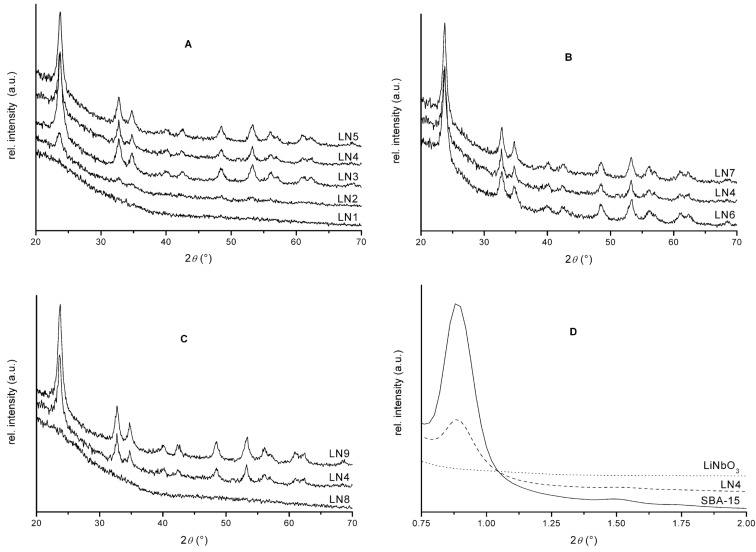
Wide-angle X-ray diffraction patterns of as-prepared LiNbO_3_/SBA-15 composites (LN). Effect of reaction temperature (A), heating rate (B), and reaction time (C). Small-angle X-ray diffraction patterns of SBA-15, composite and isolated nanoparticles (D). (For details of the experimental conditions for each sample, see Experimental section.)

The X-ray powder diffraction (XRD) patterns of the as-prepared samples were dependent on the reaction temperature, heating rate and reaction time, and showed only the reflection peaks of the hexagonal LiNbO_3_ structure (ICSD 20-631) (Figure 1A, Figure 1B and Figure 1C). As can be seen in Figure 1A, the start of crystallization can be detected at 850 °C, and at 900 °C all reflections are observed. The intensity of the reflections increases with increasing temperature which indicates a higher degree of crystallinity. The duration of heating (Figure 1B) and the reaction time (Figure 1C) are not dominant effects on the phase formation of LiNbO_3_ compared to the reaction temperature, but a reaction time of only 10 min is necessary. Broad peaks were observed for all samples suggesting that sintering of crystallites in the template was minimized. The determination of particle size, as calculated from the Scherrer equation, leads to a value of about 10 nm which corresponds well with the pore diameter of the ordered mesoporous silica SBA-15 (10.5 nm in diameter), verifying that the mesoporous silica is a useful template material for the synthesis of particles with a defined size.

Figure 1D shows the small-angle diffraction patterns of SBA-15, LiNbO_3_ composite (LN4) and LiNbO_3_ nanoparticles after removal of the template. The reflections of SBA-15 correspond to the two-dimensional hexagonal *P*6*mm* symmetry of the well ordered pore system. The XRD pattern of the impregnated material (LN4) shows a single diffraction peak of lower intensity than those of the SBA-15, which indicates that the ordered pore arrangement is not disrupted, even after the incorporation of LiNbO_3_ nanoparticles. The decrease in intensity is a consequence of the impregnation which reduces the scattering contrast between the silica walls and the empty pores. After removal of SBA-15 with LiOH solution, no peaks can be observed at low angles, which illustrates that the 2D ordered pore system - as expected - does not remain intact. Elemental analyses of these materials showed that there was no silica residue after the treatment.

**Figure 2:**
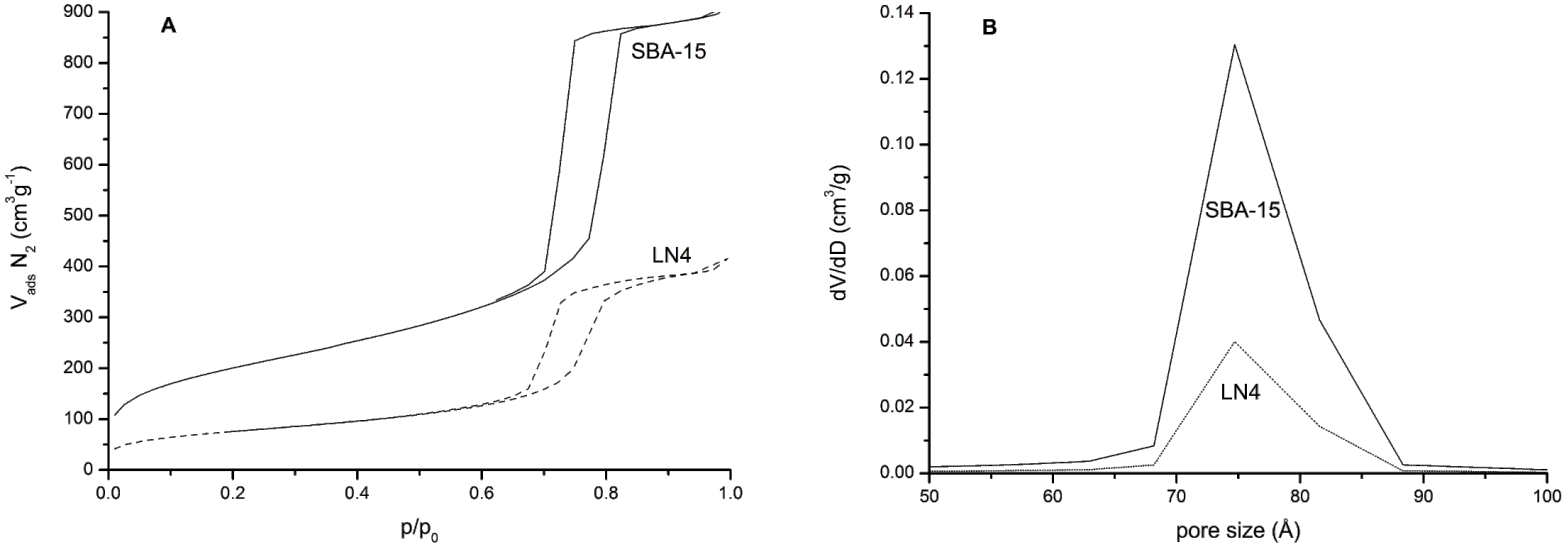
N_2_ adsorption and desorption isotherms (77 K) of SBA-15 and as-prepared LiNbO_3_/SBA-15 composite (A) and the corresponding pore size distributions of the samples.

Figure 2 shows the nitrogen physisorption isotherms of the SBA-15 and LiNbO_3_/SBA-15 composite (LN), and the corresponding pore size distributions of the samples. The measurements were recorded to investigate the effect of incorporation of LiNbO_3_ on the pore properties of the samples. The data of the specific BET surface areas and pore volumes are listed in Table 1. The textural properties of the unloaded SBA-15 when treated according to the LiNbO_3_ reaction conditions do not change significantly. For both samples, nitrogen adsorption–desorption curves show type IV isotherms with hysteresis loops, which is typical for mesoporous solids, suggesting that the ordered structure remains intact after LiNbO_3_ loading. Furthermore, the isotherms show a significant reduction of the specific BET surface area from 729 m^2^·g^−1^ for the SBA-15 to 130–160 m^2^·g^−1^ after impregnation with nanoparticles, as expected. The pore size distribution curves indicate identical pore sizes for both materials, and narrow pore size distributions in the range of 7.0–8.5 nm. These values are typical for the formation of particles inside the pore system and are in accord with the results of small-angle X-ray diffraction.

**Table 1:** Textural characterization of as-prepared LiNbO_3_/SBA-15 composites (LN).

Sample	*d*_part_^a^ (nm)	*S*_g_^b^ (m^2^·g^−1^)	*V*_p_^c^ (cm^3^·g^−1^)
SBA-15	—	729	1.38
LN2	—	151	0.39
LN3	9	158	0.38
LN4	10	138	0.30
LN5	12	131	0.32
LN6	10	158	0.33
LN7	11	160	0.36
LN9	10	213	0.44

^a^average particle diameter estimated from peak broadening; ^b^specific BET surface area; ^c^pore volume.

**Figure 3:**
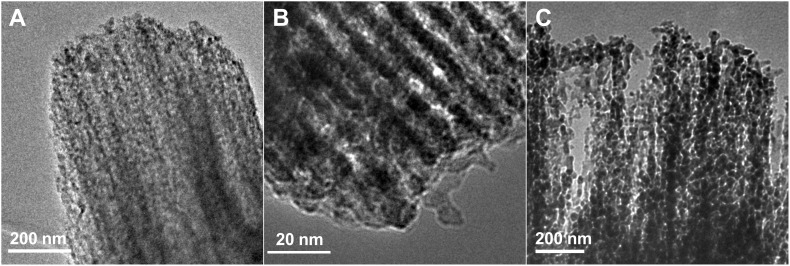
Transmission electron microscope (TEM) images of as-prepared LiNbO_3_/SBA-15 composite (A, B), and after the first treatment with LiOH solution (C).

In the TEM images of the composites (Figure 3A and Figure 3B), the ordered mesoporous structure of the silica matrix is also observed, and the nanoparticles are detected as black dots. In combination with the results from the XRD analysis, these black points can be interpreted as LiNbO_3_ nanoparticles. The particles detected as small black points are more or less homogeneously distributed throughout the entire template and the size of the LiNbO_3_ nanoparticles is comparable to the pore diameter in SBA-15. In Figure 3C the beginning of the template removal after the first treatment with LiOH solution is demonstrated. The holes in the matrix attest to the collapse of the ordered pore silica structure.

**Figure 4:**
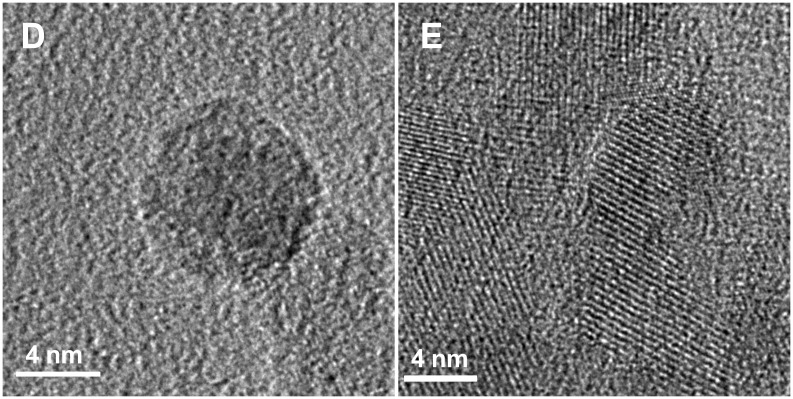
High resolution transmission electron microscope images of LiNbO_3_ nanoparticles after template removal.

The high resolution TEM images of the nanocrystalline particles (Figure 4) show spherical morphology of one isolated nanoparticle after removal of the silica matrix, and the *d*_012_ lattice spacing (*d*_012_ = 3.754 Å). This value is in agreement with the spacing measured by X-ray powder diffraction. According to the XRD particle size determination (Scherrer equation), no changes are observed for the crystallite size after complete removal of the mesoporous silica. Furthermore, chemical analyses confirm LiNbO_3_ formation, and a molar ratio of Li/Nb/O of 1/1.02/2.98.

## Conclusion

We have presented a template approach for the preparation of nanocrystalline LiNbO_3_ with grain sizes of 10 nm by using mesoporous SBA-15 silica as rigid host. The influence of the reaction temperature dominates particle size and crystallization, and a reaction time of 10 min is only necessary to obtain the phase formation in an IR furnace, while the heating rate has no significant effect. XRD patterns and other characterization results demonstrate crystallinity and phase homogeneity of all as-synthesized samples.

It is expected that the reported method could be extended to prepare other important multi-component oxides, especially ferroelectric materials.

## Experimental

**Synthesis of SBA-15** [19]**.** In a typical preparation, 4 g of Pluronic P123 was dissolved in 71.8 g of distilled water and 2.2 g of concentrated HCl (37%) with stirring for 24 h at 35 °C. Then, 7.9 g of TEOS was added and the resulting mixture stirred for 24 h at 35 °C and then stored at 130 °C for 24 h without stirring. The solid product was recovered, washed with ethanol/HCl solution and dried in an oven at 100 °C for 2 h. Calcination was carried out by slowly increasing the temperature from room temperature to 180 °C in 3 h, then maintaining the temperature at 180 °C for 3 h followed by increasing the temperature from 180 °C to 550 °C over 6 h and finally heating at 550 °C for 5 h.

**Synthesis of LiNbO_3_/SBA-15 composite.** For the precursor solution, LiNO_3_ (0.013 mol, 95%, Grüssing) and NH_4_NbO(C_2_O_4_)_2_·*x*H_2_O (0.013 mol, 99.99%, Aldrich) were mixed in a 1:1 molar ratio and added to 7 g of distilled water. After the mixture was heated for 30 min at 60 °C, all the solid material had dissolved. 1 g of the SBA-15 material was impregnated with 3.5 mL of the aqueous precursor solution by incipient-wetness method. Crystallization was carried out in air using an IR furnace IRF 10 (Behr) with a high heating rate (1–10 °C/s) to a temperature range of 750–1000 °C and heating times of between 5–20 min at these temperatures (Table 2). Finally, the calcined samples were suspended in a 2.5 M LiOH solution five times with stirring for 24 h to remove the SBA-15 matrix. The solid product was filtered, washed with distilled water until pH-neutral and air-dried at 100 °C.

**Table 2:** Reaction conditions of as-prepared LiNbO_3_/SBA-15 composites (LN).

Sample	*θ*^a^ (°C)	*r*^b^ (°C/s)	*t*^c^ (min)
LN1	750	3	10
LN2	850	3	10
LN3	900	3	10
LN4	950	3	10
LN5	1000	3	10
LN6	950	1	10
LN7	950	10	10
LN8	950	3	5
LN9	950	3	20

^a^reaction temperature; ^b^heating rate; ^c^reaction time.

**Characterization.** Wide-angle X-ray diffraction patterns were taken on a STOE Stadi-P diffractometer in transmission geometry using Cu Kα_1_ radiation (wavelength λ = 0.15405 nm). The average crystallite size was calculated from the Scherrer equation (STOE size/strain [20]). Instrumental broadening of reflections was taken into account based on LaB_6_ reference measurements. Small-angle X-ray diffraction patterns were recorded on a Bruker AXS Nanostar. The nitrogen physisorption isotherms at 77 K were measured using a Quantachrome Autosorb 1C. The samples were pre-treated at 150 °C for 3 h in vacuum (10^−6^ bar). The specific surface area was calculated from the BET (Brunauer–Emmet–Teller) equation (*p*/*p*_0_ = 0.05–0.20). The pore volume was determined at relative pressure of *p*/*p*_0_ = 0.95. The pore size distribution was estimated from the desorption branch of the isotherm using Density Functional Theory (DFT). Chemical analyses to determine the content of lithium, and niobium were carried out with an ICP-OES Vista (Varian). The samples were digested in hydrofluoric acid with microwave heating at 180 °C for 20 min. The oxygen content was determined with a TCH-600 (Leco) using the carrier gas hot extraction technique. TEM investigations were carried out with a 200 kV-TEM FEI Tecnai F20/Cs-corrected electron microscope at the Triebenberg Laboratory for high resolution TEM and electron holography.
